# Circular RNAs in tumor immunity and immunotherapy of breast cancer

**DOI:** 10.3389/fimmu.2026.1923329

**Published:** 2026-09-15

**Authors:** Yu Jiang, Ya-Wen Wang, Kai Zhang

**Affiliations:** Department of Breast Surgery, General Surgery, Qilu Hospital of Shandong University, Jinan, Shandong, China

**Keywords:** breast cancer, circular RNA, immune checkpoint, immunotherapy, tumor immune regulation

## Abstract

As the stable and conserved circular non-coding RNAs, circular RNAs (circRNAs) have assumed growing significance in regulating breast cancer immunity and immunotherapy. This review focuses on elucidating the molecular mechanisms through which circRNAs influence the efficacy of breast cancer immunotherapy. Specifically, circRNAs modulate the levels and stability of critical immune checkpoints, including PD-L1 and various cytokines. They achieve this by acting as competing endogenous RNAs (ceRNAs) to sequester miRNAs, functioning as protein scaffolds, and thereby modulating gene expression and tumor immune evasion. Furthermore, circRNAs are capable of facilitating communication between tumor cells and immune cells. In addition, it can be translated into proteins that aid in remodeling the tumor immune microenvironment. These changes may include driving the polarization of immunosuppressive M2-type macrophages, while suppressing the infiltration and functional activity of cytotoxic T cells and natural killer cells. These findings highlight the significant promise of circRNAs serving as novel biomarkers and candidate therapeutic targets. Targeting specific circRNAs is anticipated to emerge as an effective approach for enhancing the efficacy of current immune checkpoint inhibitors, potentially helping to overcome drug resistance encountered in immunotherapy. Furthermore, it offers potential for the development of novel circRNA-based vaccines. Collectively, these advancements pave the way for improving the prognosis of breast cancer immunotherapy and open new avenues for future research and clinical translation.

## 1 Introduction

As a leading cause of mortality in female cancer patients, breast cancer represents the most frequently diagnosed malignant neoplasm across the globe (1). It is a heterogeneous disease (2) classified into several types, including luminal A, luminal B, HER2-positive, and triple-negative breast cancer (TNBC). Due to the diversity of subtypes, the treatment methods for breast cancer vary (3–5). The principal therapeutic approaches encompass surgical intervention, hormone-based treatment, cytotoxic chemotherapy (including neoadjuvant regimens), radiation therapy, and precision-targeted agents. For steroid hormone receptor-positive breast cancer, including luminal A and luminal B types, endocrine therapy can achieve favorable outcomes. Targeted therapy is primarily used for HER2-positive breast cancer. In contrast, TNBC lacks corresponding molecular targets, and the current treatment methods are associated with limited efficacy and a poor prognosis (6, 7).

Immunotherapy is a strategy that modifies immune responses—whether through regulation, enhancement, or reconstitution—to utilize the selective recognition, long-term memory, and adaptability of immune system in combating infections, neoplasms, or aberrant immune states, thereby enabling disease control. In recent years, immunotherapy has profoundly changed the landscape of tumor treatment (8, 9). Key strategies within immunotherapy principally comprise immune checkpoint inhibitors, engineered cell transfer therapies, tumor-targeted vaccines, and viruses designed to selectively lyse cancer cells. Immune checkpoints refer to specific proteins displayed on immune cells or tumor cells that modulate immune responses. Through ligand-receptor engagement, these molecules govern immune cell activation, growth, and differentiation, thereby maintaining immune homeostasis, regulating autoimmune pathologies, and enabling tumor cells to evade immune detection (10, 11). PD-1/PD-L1 is a classic immune checkpoint, which plays a rate-limiting role in anti-tumor immune responses and promotes tumor immune escape (12). Immune checkpoint inhibitor therapy aims to neutralize the suppressive signals exerted by immune checkpoints on effector cells, thereby reactivating immune cells to eradicate cancer cells. This therapeutic approach has suppressed tumor proliferation in non-small cell lung cancer and malignant melanoma, and improved patient overall survival (13). Adoptive cell therapy involves extracting a patient’s immune cells, genetically modifying or expanding them *in vitro*, and subsequently reinfusing the activated cells to target and eliminate tumors. It mainly includes chimeric antigen receptor T-cell therapy (CAR-T), T cell receptor-engineered T cell therapy, NK cell therapy, macrophage cell therapy, cytokine-induced killer cell therapy, and regulatory T cell therapy (14–16). Clinical application of CAR-T therapy has centered on hematologic cancers. However, its impact on solid tumors is reduced by T-cell exhaustion, brief duration of response, and off-target adverse events. Yet, studies have found that circular RNAs (circRNAs) can reduce this inhibition, regulate the tumor microenvironment, and enhance the therapeutic specificity and efficacy of CAR-T therapy in solid tumors (17). Tumor vaccines, which concentrate on​ tumor-associated antigens or tumor-specific antigens to strengthen immune recognition and cytotoxicity against tumor cells harboring particular antigen markers, have also achieved improved results in experimental studies (18). Oncolytic viruses possess the c-apacity to selectively and directly rupture cancer cells, while also indirectly bolstering antitumor immune responses through the discharge of tumor antigens and induction of inflammatory signaling within the tumor microenvironment (19). Oncolytic virotherapy has emerged as a prominent immunotherapeutic modality, demonstrating robust tumor-inhibitory effects across diverse malignancies including breast cancer (20).

Immunotherapy has emerged as a critical strategy in breast cancer, particularly for aggressive subtypes like TNBC, where the complex interplay within the tumor microenvironment presents both a challenge and an opportunity for therapeutic intervention (21). Pembrolizumab, a PD-1 immune checkpoint inhibitor, has been approved by the U.S. Food and Drug Administration for the treatment of TNBC (22). Oncolytic viruses can reshape the tumor microenvironment to exert antitumor effects in breast cancer. It is worth emphasizing that, at present, the evaluation of oncolytic viral agents against breast carcinoma in terms of antitumor potency and safety is still limited to preclinical phases (23, 24). Findings from a single-arm phase 2 clinical trial signal that combining oncolytic virotherapy with neoadjuvant chemotherapy may augment therapeutic response in patients with high-risk early-stage TNBC. However, adverse events attributable to oncolytic virotherapy—including pyrexia, chills, headache, fatigue, and injection-site pain—require refined safety management protocols and necessitate further dedicated investigation (25). CAR-T therapy suppresses breast cancer progression by engaging multiple targets. Nonetheless, its broader clinical implementation is constrained by​ persistent risks of off-target effects and cytokine release syndrome (26). Tumor vaccines have been tested in multiple breast cancer subtypes, owing to their intrinsic capacity to prime antitumor effector cells and reawaken immune surveillance. However, clinical validation to date has been circumscribed, with human trials reporting only marginal gains over standard-of-care regimens (27). Currently, breast cancer is characterized by the profound heterogeneity, a paucity of tumor-specific antigens, and an immunosuppressive microenvironment. Hence, identifying key new targets for immunotherapy, investigating the mechanisms of action, and exploring novel immunotherapeutic drugs are poised to emerge as primary focuses for improving breast cancer management (28).

Circular RNAs are covalently closed circular endogenous non-coding biomolecules in eukaryotes. They show spatially restricted expression, limited to specific tissues and cell populations, and their biosynthesis is regulated by specific cis-acting elements and trans-acting factors. These molecules are evolutionarily conserved and abundantly and stably expressed in cells (29). Moreover, circRNAs carry out diverse cellular functions, encompassing acting as decoys for microRNAs, serving as a structural framework for protein complexes, engaging in protein binding, coding for peptides, and modulating transcriptional or post-transcriptional output (30). The presence and function of circRNAs are closely linked to tumorigenesis and subsequent growth, as they are involved in assorted biological controls of the tumor niche and offer strong prospects for oncologic interventions (31, 32). CircRNAs affect the expression and function of various molecules, including immune molecules and immune targets. The development of circRNA-based targeting strategies holds promise for increasing the potency of immunotherapy in the treatment of breast cancer (33). This review summarizes the roles and molecular mechanisms of circRNAs in breast cancer, and their implications in immunotherapy.

## 2 The molecular mechanisms and roles of circular RNAs in breast cancer

### 2.1 Biogenesis, classification and characteristics of circular RNAs

Early studies considered circRNAs as by-products generated by abnormal splicing during linear RNA synthesis (34). However, recent findings show that the biogenesis of circRNAs follow a precise process, involving the back-splicing of precursor mRNAs (35). Through the coordinated action of cis-regulatory sequences and trans-acting proteins, the downstream 5’ splice site of an exon links to the upstream 3’ splice site, leading to circRNA biogenesis (36–38). For example, exon circularization depends on complementary sequences in the flanking introns. And the alternative formation of inverted repeat Alu pairs and the competition among them can lead to alternative circularization, thereby enabling a single gene to generate multiple circular RNA transcripts (39). CircRNAs are dynamically regulated by Quaking, an alternative splicing factor that also functions as a trans-acting factor. Moreover, circRNA abundance is modulated by intronic Quaking-binding motifs (40). Moreover, circRNAs may exert regulatory functions in gene expression through competition with linear splicing (41). It follows that circRNAs can be circularized via diverse pathways, giving rise to multiple circular RNA transcripts.

Based on their biogenesis and genomic composition, circRNAs are classified into several distinct categories. The most prevalent type, exon-derived circRNAs (ecircRNAs), are primarily generated through three well-characterized mechanisms: lariat-driven circularization, intron-pairing-driven circularization, and resplicing-driven circularization (42). A second specialized class comprises exon-intron circular RNAs (EIciRNAs). Unlike ecircRNAs, EIciRAs retain both exonic and intronic sequences, generated through two mechanisms: intron pairing drive and lasso drive, enabling them to function in both the nucleus and the cytoplasm (43, 44). Their core function is to promote the transcription of its maternal gene in cis (44). Additionally, intron-derived circRNAs (ciRNAs) represent another significant subgroup. Composed solely of intronic sequences, these RNAs play pivotal roles in regulating gene expression (37, 45). For instance, ciRNAs may form complexes with RNA-binding proteins, which subsequently associate with pre-mRNA transcripts to modulate their stability, thereby ultimately affecting gene expression (46). Such structural differences give rise to variations in their subcellular localization and stability. Specifically, EIciRNAs and ciRNAs typically remain in the nucleus after biogenesis, whereas ecircRNAs are transported to the cytoplasm and exhibit greater stability (47).The absence of 5’ and 3’ ends in their unique circular structure endows circRNAs with high stability. The circular structure of these molecules shields them from cleavage by RNA exonucleases or RNases, unlike linear RNAs (48–50). Moreover, the relatively low immunogenicity​ of certain circRNAs positions them as promising candidates​ for therapeutic development (51). CircRNAs exhibit remarkable cell-type- and tissue-specific expression patterns (52). Furthermore, circRNAs can be packaged into exosomes and secreted into the extracellular milieu, where they function as crucial mediators​ in modulating signaling pathways, the tumor microenvironment, and immune responses (53).

### 2.2 Mechanisms of action of circular RNAs in breast cancer

Advances in high-throughput RNA sequencing have enabled the discovery of a growing list of circRNAs. An increasing number of studies have found that circRNAs exert impacts on various biological processes through multiple pathways, including cell proliferation, cell migration, metabolism, and immunity (54). Furthermore, aberrant levels of circRNAs are strongly implicated in breast cancer onset and development, acting via diverse modes of action to either foster or restrain disease progression (32).

### 2.2.1 Competitive miRNA sponging

Among the key mechanisms of circRNA action is their role as competing endogenous RNAs (ceRNAs) that act as sponges for miRNAs, influencing the interaction of mRNAs with downstream gene targets (55–57). In TNBC, downregulated circFBXW7 acts as a molecular sponge for miR-197-3p. CircFBXW7 suppresses the growth and metastasis of TNBC by sequestering miR-197-3p and subsequently upregulating FBXW7 expression (58). In breast cancer, circ_0025202 shows diminished expression, hinders disease advancement through regulation of the miR-182-5p/FOXO3a pathway, and augments the responsiveness to tamoxifen resistance (59). Dysregulated circBACH2 in breast cancer interacts with N6-methyladenosine (m^6^A)-modified hsa-miR-944, raising HNRNPC expression and driving cell proliferation through activation of the MAPK signaling cascade (60). The higher circPSMA1 abundance in TNBC cells and in exosomes present in patient serum. CircPSMA1 sponges miR-637, thereby affecting –its target gene Akt1, as well as the downstream target genes β-catenin and cyclin D1, promoting the proliferation, metastasis and immune suppression of TNBC cells (61).

In essence, the dynamic interplay between circRNAs and miRNAs constitutes a critical regulatory axis in breast cancer pathogenesis, promoting tumor progression, drug resistance, and immunosuppression, thus highlighting this axis as a promising therapeutic target.

### 2.2.2 Protein scaffolding

CircRNAs can function as protein scaffold and facilitate intermolecular interactions. Specifically, they exert this function by recruiting binding proteins, tethering and transporting proteins to other subcellular sites, and modulating protein-protein interactions (57, 62–64). CircRNA-CREIT bridges PKR to the E3 ligase HACE1, thereby orchestrating K48-linked polyubiquitylation that earmarks PKR for proteasomal degradation. Ultimately, this cascade sensitizes TNBC cells to doxorubicin (65). Hypoxia-triggered circWSB1, which exhibits aberrant expression in breast cancer, competes with p53 for binding to USP10, attenuating USP10-mediated stabilization of p53, enhancing p53 degradation, and thereby promoting tumor progression (66). CrVDAC3 is overexpressed in breast cancer and binds HSPB1 via several sites, preventing ubiquitin-mediated breakdown of HSPB1 and elevating its expression, which mitigates ferroptosis and promotes trastuzumab resistance in HER2-low-expressing cases (67). CircEGFR facilitates ANXA2 translocation to the plasma membrane in TNBC cells, which subsequently releases TFEB from the ANXA2-TFEB complex, allows TFEB nuclear import, and ultimately promotes autophagy (68).

Collectively, these findings establish that circRNAs, by functioning as scaffolds to regulate protein expression and function, dictate key malignant phenotypes.

### 2.2.3 Template-directed protein translation

Due to their special circular structure-lacking 5’’ and 3’’ends-circRNAs have traditionally been classified as non-coding RNA molecules. However, recent research has shown that circRNAs can translate certain proteins or polypeptides either through elements such as internal ribosome entry sites, open reading frames, or via m^6^A-mediated mechanisms (69–72). CircFBXW7 is capable of encoding a 21 kDa peptide, designated FBXW7-185aa, which downregulates c-Myc expression and suppresses the advancement of TNBC (58). Circ-EIF6 gives rise to EIF6-224aa, which binds MYH9 directly, hampers ubiquitin-mediated breakdown, boosts MYH9 expression, activates the Wnt/β-catenin pathway, and thereby accelerates TNBC cell proliferation and metastatic potential (73). CircTRIM1 can translate the protein TRIM1-269aa through functional IRES elements and ORFs. The protein augments the binding of MARCKS to calmodulin, driving calmodulin-mediated delocalization of MARCKS, consequently driving the activation of the PI3K/AKT/mTOR signaling cascade and promoting both chemoresistance and metastatic progression in TNBC (74).

Beyond their structural uniqueness, circRNAs harbor cryptic ORFs capable of encoding bioactive peptides that either suppress or promote tumorigenesis, highlighting their dual potential as both biomarkers and therapeutic targets​ in breast cancer management.

### 2.2.4 Transcriptional regulation

Studies have found that EIciRNAs can bind to small nuclear RNAs (snRNAs) through RNA-RNA interactions. The interplay of EIciRNAs with U1 snRNA can promote the binding of EIciRNAs to U1 small nuclear ribonucleoprotein (U1 snRNP). The EIciRNA-U1 snRNP complex further interacts with the RNA polymerase II transcription complex at the promoter of the parental gene of EIciRNAs, leading to increased gene expression (44). In the hormone receptor-positive breast cancer cell line MCF-7, the back-splicing site of circHIPK3 interacts with the last coding exon of *BRCA1* mRNA. This interaction competitively relieves the inhibitory effect of FMRP on *BRCA1* mRNA translation, promotes the expression of BRCA1 protein, reduces DNA damage, and diminishes the sensitivity of breast cancer cells to DNA damage inducers (75) CircSMARCA5 engages its parental *SMARCA5* locus in an R-loop that sterically occludes Pol II recruitment, extinguishing *SMARCA5* transcription. Since SMARCA5 safeguards genomic integrity and directs DNA damage repair, derepression of the *SMARCA5* gene following circSMARCA5 downregulation in breast cancer confers heightened repair capacity, sustained genome stability, and marked cisplatin insensitivity (76).

Together, these data highlight that circRNAs function as pivotal modulators​ by engaging in specific base-pairing interactions with snRNAs or cognate mRNAs, thereby orchestrating chromatin dynamics and gene expression in breast cancer (**Figure 1**).

**Figure 1:**
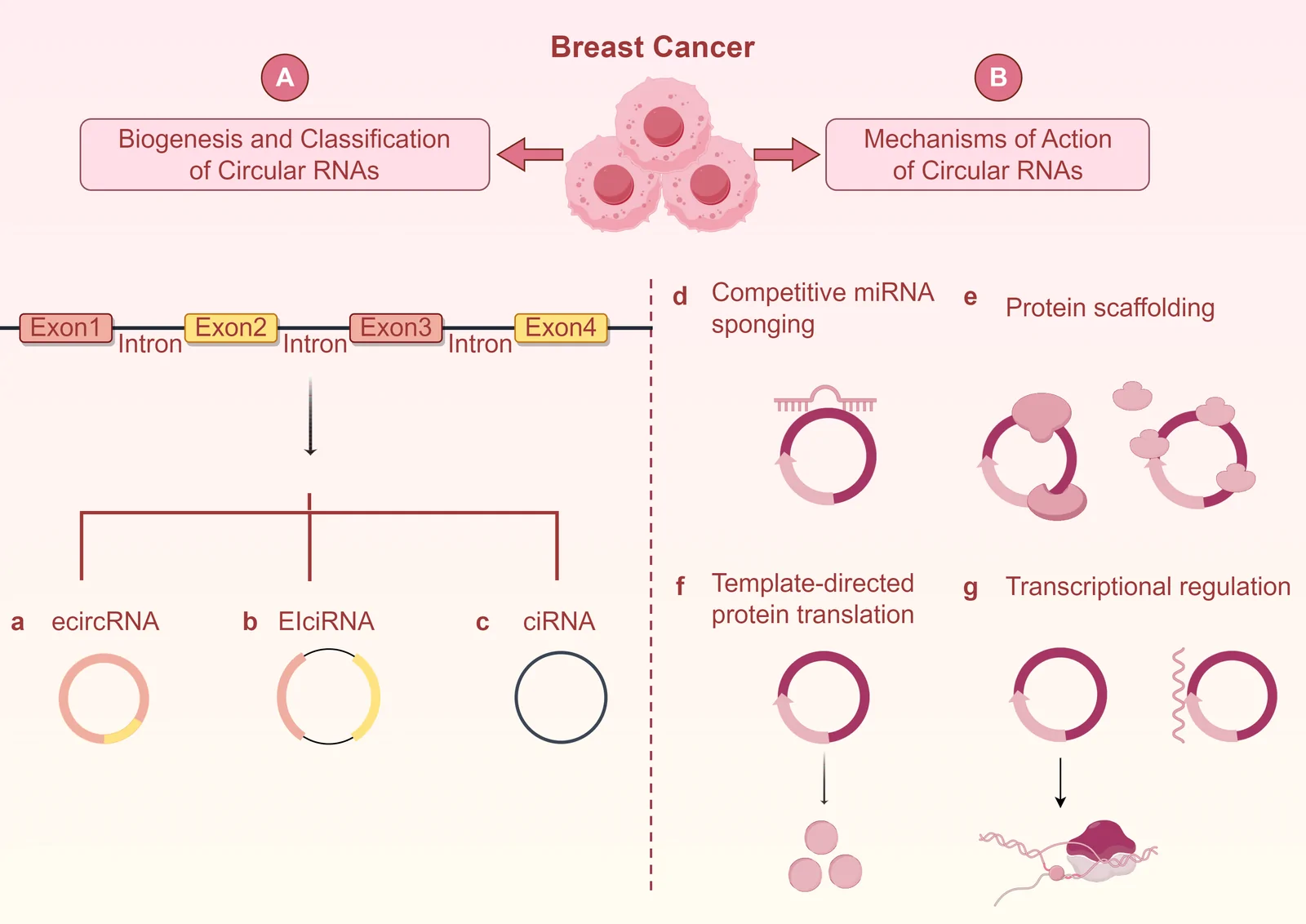
The biogenesis, classification and functional mechanism of circular RNAs in breast cancer. **(A)** The biogenesis, classification of circular RNAs. **(a)** ecircRNA. **(b)** EIciRNA. **(c)** ciRNA. **(B)** The mechanisms of action of circular RNAs. **(d)** Circular RNAs act as a molecular sponge to bind with miRNAs. **(e)** Circular RNAs act as a protein scaffold or protein decoy. **(f)** Circular RNAs act as a template for translating proteins. **(g)** Circular RNAs act as a regulator influencing gene expression.

### 2.3 Biological roles of circular RNAs in breast cancer

### 2.3.1 Occurrence of breast cancer

The initiation of breast carcinogenesis is frequently rooted in the dysregulation of connexin-mediated intercellular communication and the subsequent loss of epithelial polarity. Previous studies have established that silencing the Cx43 protein can disrupt cell stratification and polarity, thereby simulating the precancerous state of the breast (77). Hsa_circ_0077755 originates from the Cx43 gene and is expressed at lower levels. It affects miR-182 through the molecular sponge mechanism, and is related to abnormal changes in the polarity of mammary epithelial cells and in tumor-related pathways. Therefore, it is believed that the Cx43/hsa_circ_0077755/miR-182 regulatory axis may function as a post-transcriptional biomarker panel for evaluating breast cancer susceptibility (78).

### 2.3.2 Progression of breast cancer

CircRNAs have emerged as pivotal modulators of cellular plasticity and organotropism. Moreover, as part of a complex network of transcriptional regulators, they orchestrate the progression and metastatic cascade in breast cancer. Specifically, circRPPH1, which is highly expressed in breast cancer, is associated with advanced breast cancer stages and poor prognosis. It accelerates the cell cycle of TNBC cells, promotes cell proliferation, metastasis and invasion, and inhibits cell apoptosis (79). CircBCBM1 is overexpressed in breast cancer cells, where it facilitates their trans-endothelial migration and invasion across the blood-brain barrier through the circBCBM1/miR-125a/BRD4/MMP9 axis, enhancing brain metastasis (80). In breast cancer, circLIFR-007 binds to hnRNPA1 and promotes the transfer of hnRNPA1 from the nucleus to the cytoplasm. After hnRNPA1 dissociates from circLIFR-007 in the cytoplasm, it binds to the YAP, increasing the phosphorylation level of YAP protein, thereby inhibiting the transcriptional regulation of specific liver metastasis-related protein factors after YAP enters the nucleus, and ultimately inhibiting the liver metastasis of breast cancer (81).

### 2.3.3 Tumor markers for breast cancer

The inherent stability of circRNAs in biofluids and their dysregulated expression profiles position them as promising non-invasive biomarkers for breast cancer management. In TNBC, circSEPT9 is markedly upregulated and correlates with more advanced primary tumor size, nodal involvement, and overall TNM classification. Elevated circSEPT9 levels are linked to reduced overall survival in TNBC patients, indicating its potential as an independent biomarker for diagnosis and prognosis (82). High expression of CircROBO1 in breast cancer is linked to enhanced tumor progression, spread to the liver, and poor prognosis (83). CircTBPL1, abundantly encapsulated in exosomes secreted by cancer-associated fibroblasts, functions as a molecular sponge for specific miRNAs. Owing to its cancer-associated fibroblasts-specific enrichment within the exosomal compartment, circTBPL1 represents a compelling non-invasive liquid biopsy biomarker for early diagnosis, prognostic stratification, and monitoring of therapeutic response in breast cancer (84).

### 2.3.4 Therapeutic targets for breast cancer

The functional diversity of circRNAs positions them as highly tractable therapeutic vulnerabilities in breast cancer, offering avenues to overcome the multifaceted challenge of treatment resistance. Evidence indicates that irradiation leads to downregulation of hsa_circ_0001610 in breast cancer cells. Treatment with dihydroartemisinin suppresses this circRNA, triggering ferroptosis via the hsa_circ_0001610/miR-139-5p/SLC7A11 axis and thereby increasing radiosensitivity (85). CircDUSP16 exhibits high expression across breast cancer cells, tumor tissues, and plasma-derived exosomes from patients, where it drives proliferation, metastasis, and invasion via the miR-1224-3p/TFDP2 signaling axis. Nevertheless, this oncogenic effect of circDUSP16 can be inhibited by pirarubicin, a second-generation anthracycline chemotherapy drug. Targeting circDUSP16 may affect the therapeutic effect of pirarubicin (86). Circ-β-TrCP is highly expressed in trastuzumab-resistant HER2-positive breast cancer and can encode the protein β-TrCP-343aa. This protein inhibits the SCFβ-TRCP-mediated proteasomal degradation pathway of NRF2 through direct binding to NRF2. The increased level of the basic leucine zipper transcription factor NRF2 upregulates the expression of a set of antioxidant genes at the transcriptional level, promoting trastuzumab resistance (87).

In summary, circular RNAs play critical roles in the occurrence, progression, diagnosis, and treatment of breast cancer (**Figure 2**).

**Figure 2:**
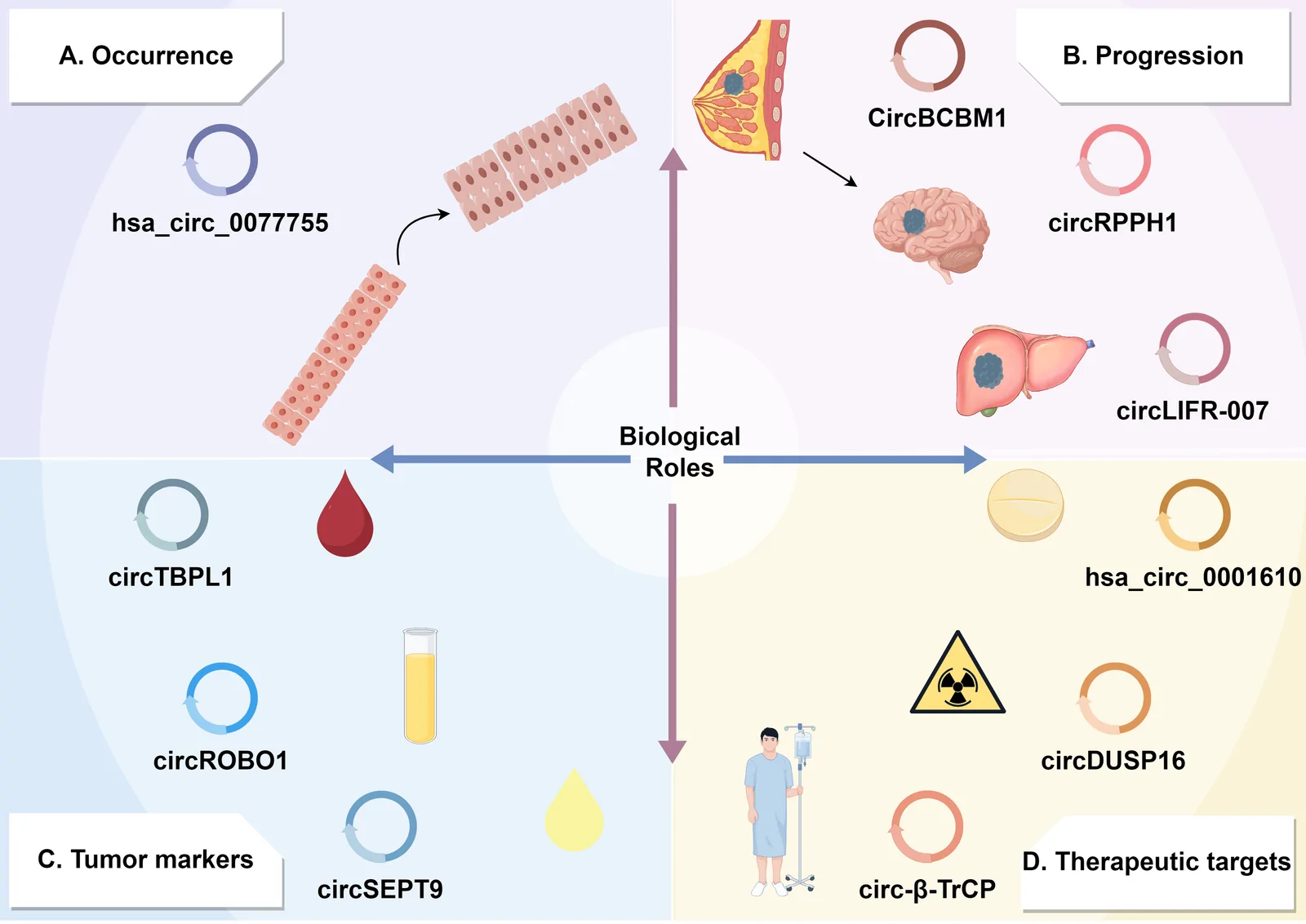
The biological roles of circular RNAs in breast cancer **(A)** Circular RNAs promote the occurrence of breast cancer. **(B)** Circular RNAs promote the progression of breast cancer. **(C)** Circular RNAs act as tumor markers for breast cancer. **(D)** Circular RNAs act as therapeutic targets for breast cancer.

## 3 The roles and mechanisms of circular RNAs in breast cancer immunity and immunotherapy

Alongside standard therapies—including surgery, chemotherapy, and radiation—the significant impact of immunotherapy on breast cancer management has garnered growing interest. Evidence from clinical investigations has demonstrated that immunotherapy enhances overall survival and lowers mortality in breast cancer (28). Current explorations in immunotherapy for breast cancer mainly include immune checkpoint blockade, CAR-T-cell immunotherapy, tumor vaccines, and oncolytic viruses. Given the multifaceted immune system and patient-specific differences, along with tumor heterogeneity in breast cancer, immunotherapy is subject to notable limitations (88). The modification of tumor antigens, gene mutations, changes in tumor-infiltrating lymphocytes and some immune molecules regulate the tumor microenvironment with dynamic heterogeneity and immunosuppressive properties, which diminishes the responsiveness of breast cancer to immunotherapy and fosters drug resistance (13, 89). It is necessary to explore new molecules that regulate immune pathways, and activate the immune system by increasing or decreasing their expression, so as to inhibit development of breast cancer and reduce drug resistance.

In recent years, growing data indicate that circRNAs are critically involved in diverse facets of breast cancer immunity, including modulation of immune checkpoint pathways, regulation of immune cells, and cytokines, along with substantial influences on tumor immunotherapy outcomes (90, 91). Immunoregulation of circular RNAs in breast cancer is summarized in **Table 1**.

**Table 1:** Immunoregulation of circular RNAs in breast cancer.

CircRNA	Tumor subtyping	Dysregulation in breast cancer	Experimental model	Molecular target	Function mechanism	Regulated immune pathways/Cells/cytokines	Therapeutic significance	Reference
circ-0000512	TNBC	Up	*In vitro* and *in vivo*	miR-622	ceRNA	PD-L1/PD-1	TNBC growth and immune escape	(92)
circGSK3β	BC	UP	*In vitro*	miR-338-3p	ceRNA	PD-L1/PD-1	Progression and immune evasion	(93)
hsa_circ_0001598	HER2-positive BC	Up	*In vitro*	miR-1184	ceRNA	PD-L1/PD-1	Progression and immune evasion Trastuzumab resistance	(94)
circFGFR4	TNBC	Up	*In vitro* and *in vivo*	miR-185-5p	ceRNA	PD-L1/PD-1	Progression Resistance to anti-PD-1 immunotherapy	(95)
hsa_circ_0067842	BC	Up	*In vitro*	HuR	Protein scaffolding	PD-L1/PD-1	Metastasis and immune escape	(96)
circCFL1	TNBC	Up	*In vitro* and *in vivo*	HDAC1/c-Myc	Protein scaffolding	PD-L1/PD-1	Progression, stemness and immune escape	(97)
circATAD2	BC	Up	*In vitro* and *in vivo*	m^6^A/IGF2BP3/*PD-L1* mRNA	Transcriptional regulation	PD-L1/PD-1	Progression and immune evasion	(98)
circFAM53B	BC	Up	*In vitro* and *in vivo*	cryptic peptide	Template-directed protein translation	CD8+ T cell	Better prognosis Peptide vaccines and circRNA vaccines	(99)
circ_002172	BC	UP	*In vitro* and *in vivo*	miR-296-5p	ceRNA	cytotoxic T lymphocytes	Progression and immune escape	(100)
circ_0001142	BC	UP	*In vitro* and *in vivo*	miR-361–3p	Exosome/ceRNA	M2 polarization	Proliferation, migration, cell cycle and apoptosis	(101)
cSERPINE2	BC	UP	*In vitro* and *in vivo*	miR-513a-5p	Exosome/ceRNA	TAM	Progression PLGA-PEG (si-cSERPINE2) NP	(102)
circCLASP1	BC	UP	*In vitro* and *in vivo*	CCT2	Protein scaffolding	M2 polarization	Progression, immunosuppressive microenvironment and lung metastasis	(103)
circ_0076611	TNBC	UP	*In vitro* and *in vivo*	STAT6 signal	Exosome/Not Determined	M2 polarization	Growth, lung metastasis and the therapeutic drug	(104)
circSpdyA	TNBC	UP	*In vitro* and *in vivo*	127aa	Template-directed protein translation	NK cell	Metabolic alterations and tumorigenesis	(105)
circTNK2	ER-positive BC	UP	*In vitro* and *in vivo*	STAT3/SRSF1	Protein scaffolding	NK cell	Growth Tamoxifen resistance	(106)
circWWC3	BC	Up	*In vitro*	Not Determined	Not Determined	IL-4	Progression, migration and immune escape	(107)
circPVT1	ER-positive BC	Up	*In vitro* and *in vivo*	MAVS/miR-181a-2-3p	Protein scaffolding/ceRNA	type I IFN	Tumorigenesis Tamoxifen resistance	(108)
circPARPBP	TNBC	Up	*In vitro* and *in vivo*	NF2-related CBP activator protein complex	Protein scaffolding	CCL20	Progression Chemoresistance	(109)
circRHBDD1 (4, 5)	Luminal, HER2-positive, or basal-like breast cancer subtypes	Up	*In vitro* and *in vivo*	*CCL2* mRNA	Transcriptional regulation	CCL2	Bone metastasis	(110)

### 3.1 CircRNAs engage in the regulation of anti-PD-L1 immunotherapy in breast cancer

PD-1 and PD-L1 belong to the CD28 and B7 families, respectively. Both are classic immune checkpoints. The PD-1/PD-L1 interaction blocks T cell receptor signaling, limits clonal expansion and differentiation, triggers T cell exhaustion programs, and creates an immunosuppressive tumor milieu that impedes immunotherapy (111–113). In TNBC, circ-0000512 is overexpressed and sequesters miR-622, thereby relieving repression of CMTM6. Elevated CMTM6 expression reduces ubiquitin-mediated degradation of PD-L1, leading to increased PD-L1 expression that facilitates immune evasion and compromise the efficacy of anti-PD-L1 immunotherapy (92). Liang et al. (93) explained that circGSK3β is upregulated in breast cancer tissues, where it competitively binds to miR-338-3p and releases *PRMT5* mRNA inhibited by miR-338-3p. PRMT5 accumulates in the PD-L1 promoter region and dictates PD-L1 expression through H3K4me3, while affecting the number of T cells and tumor-killing function, thereby promoting the progression and immune escape of breast cancer. Hsa_circ_0001598 is significantly upregulated in trastuzumab-resistant breast cancer cell lines. The dysregulation of the hsa_circ_0001598/miR-1184/PD-L1 signal enhances the invasiveness by promoting breast cancer cell proliferation, acquisition of trastuzumab-resistant phenotype, and evasion of immune surveillance (94). CircFGFR4, which is elevated in TNBC tissues, competitively binds to miR-185-5p, inhibiting the binding of miR-185-5p to the 3’ untranslated region of *CXCR4* mRNA, thereby increasing the expression level of CXCR4. The study also found that high levels of circFGFR4 and CXCR4 significantly reduced the potency of anti-PD-1 therapy (95).

Hsa_circ_0067842 binds to HuR within breast cancer, modulating HuR nuclear-to-cytoplasmic shuttling. Cytosolic HuR stabilizes CMTM6, and limits PD-L1 ubiquitination and degradation, ultimately fostering tumor immune escape and metastasis (96). In breast cancer, circCFL1 can reinforce the interaction between HDAC1 and c-Myc, thereby protecting c-Myc from deacetylation-associated K48-linked ubiquitination and enhancing its stability. Stabilized c-Myc binds directly to the *TP53* promoter, upregulating mutp53 expression in *TP53*-mutant TNBC cells. Elevated mutp53 increases PD-L1 levels and suppresses CD8^+^ T cell-mediated antitumor immunity, ultimately driving immune escape in TNBC (97).

Previous work has suggested that m^6^A modification of circATAD2 in breast cancer drives its overexpression, resulting in elevated *PD-L1* mRNA levels via m^6^A alteration of the 3’ untranslated region. This modification can be recognized by the m^6^A reader IGF2BP3, consequently increasing *PD-L1* mRNA stability, leading to elevated expression, inhibiting the tumor-killing ability of CD8+ T cells, and facilitating the evasion of immune surveillance (98).

In summary, circRNAs govern PD-L1 abundance in breast cancer through different pathways which collectively drives immune evasion and confer resistance to immune checkpoint blockade.

### 3.2 Circular RNAs affect immune cells in breast cancer

T cells are the main component of adaptive immunity (114). Studies have shown that tumor-specific circFAM53B is capable of translating a concealed antigenic peptide via a non-coding mechanism, allowing it to associate with HLA-I. The cryptic peptide effectively activates naïve CD4+ T and CD8+ T cells in an antigen-specific manner. Mechanistically, the circFAM53B-derived peptide displays robust binding to both HLA class I and class II molecules. *In vivo* data indicate that vaccines incorporating tumor-specific circFAM53B or its peptide enhance cytotoxic T cell recruitment into tumors and promote efficient tumor containment (99). Circ_002172 sponges miR-296-5p, relieving restriction on *CXCL12* mRNA, thereby elevating CXCL12 levels (100). In breast cancer, CXCL12 suppresses the infiltration of cytotoxic T lymphocytes and facilitates immune evasion (115).

Advanced bioinformatics pipelines, coupled with high-throughput sequencing technologies, have proven instrumental in deciphering the intricate interplay​ between circRNAs and the tumor-immune microenvironment. For example, Taleghani et al. (116) found that in different breast cancer subtypes, circular RNAs such as circ_000349, circ_003996, circ_003925, circ_002665, and circ_001855 contribute to T cell activation, proliferation, and programmed cell death, modulate immune responses, and consequently influence breast cancer initiation and progression. Taken together, these circRNAs may serve as targets for immunotherapy. By analyzing the exosomal circRNA expression pattern, exploring differentially expressed circRNAs, and constructing a circRNA-miRNA-mRNA network, Mao et al. observed that this network is related to various immune cells in breast cancer, which may engage in immune regulation and affect the progression and prognosis of breast cancer (117).

Macrophages, present in large numbers within the tumor microenvironment, are categorized into M1 and M2 subtypes.​ M1 phenotype macrophages exert anti-tumor functions, while M2 phenotype macrophages promote tumor occurrence and development (118, 119). Overexpressed in breast cancer, circ_0001142 elevates IL-4 transcription to drive M2 macrophage recruitment and polarization. Tumor-derived exosomal circ_0001142 is further internalized by macrophages, sponges miR-361-3p to derepress PIK3CB, consolidates the M2 phenotype of macrophages, and ultimately fuels malignant progression and metastasis (101). Elevated tumor-derived exosomal cSERPINE2 is delivered to tumor-associated macrophages (TAMs), where it sponges miR-513a-5p to derepress MALT1/NF-κB signaling and drive IL-6 output. IL-6 then activates JAK2-STAT3 to upregulate EIF4A3, which sustains cSERPINE2 biogenesis and CCL2, forming a feed-forward loop that amplifies TAMs infiltration and drives breast cancer progression and metastasis (102). CircCLASP1 is elevated in breast cancer and governed by U2AF2-directed back-splicing. It stabilizes GLI1 via CCT2 tethering and drives GLI1 nuclear trafficking to induce SNAIL and CCL2/CCL5 production, promoting M2-TAM polarization, lung metastasis and tumor immune evasion. This cascade as a circCLASP1-CCT2/GLI1/SNAIL circuit that couples splicing control to TME remodeling, positioning circCLASP1 as a tractable prognostic and therapeutic candidate for breast cancer (103). Exosomal circ_0076611 promotes M2 phenotype macrophage polarization through the TRAF3IP2/STAT6 signaling axis, which in turn drives TNBC invasion. Notably, a baicalin nano-formulation disrupts the circ-TRAF3IP2 interaction, reversing these effects and reducing M2 phenotype macrophage infiltration, tumor growth, and metastasis. This indicates that circ_0076611 is a druggable target amenable to baicalin-based therapy (104).

NK cells represent a vital element of the innate immune system and contribute to tumor immune surveillance (120–122). The upregulated circSpdyA can translate a protein named 127aa. 127aa promotes FASN-mediated *de novo* synthesis of fatty acids by directly binding to FASN, increasing the fatty acid levels in TNBC. Accumulated fatty acids are transferred from TNBC to NK cells through a paracrine pathway, which inhibits the cytotoxic capacity of NK cells. Additionally, DNA methyltransferases are recruited to the vicinity of the gene promoters of NK cell activation factors, regulating DNA methylation, affecting the transcriptional activator expression and activity, and impairing the normal function of NK cells (105).CircTNK2 can encode a C-TNK2-487aa protein. C-TNK2-487aa interacts with the transcription factor STAT3, promoting STAT3 phosphorylation and nuclear translocation of phosphorylated STAT3. Once phosphorylated STAT3 translocates into the nucleus, it binds STAT1 instead of allowing STAT1 homodimerization. This reduces STAT1 binding to the CXCL10 promoter. Consequently, CXCL10 expression driven by STAT1 is lowered. Impaired CXCL10 production hinders NK cell recruitment to the tumors. The lack of NK cell infiltration supports immune escape by breast cancer cells (106).

CircRNAs critically reshape the breast cancer immune microenvironment by modulating the functional dynamics of innate and adaptive immune cells, thereby driving malignant progression and dictating therapeutic responsiveness.

### 3.3 Circular RNAs regulate cytokines interacting with immune cells

Cytokines are a class of small proteins or polypeptides secreted by immune cells or non-immune cells. They play an important role in the regulation of innate and adaptive immunity through autocrine, paracrine, or endocrine modes. Cytokines, along with tumor cells, immune cells, stromal cells, blood vessels, and extracellular matrix components, constitute a dynamic tumor microenvironment, which can regulate tumor progression, metastasis, and drug resistance (123, 124).Elevated circWWC3 levels stimulate IL-4 production and secretion in breast cancer. IL-4 stimulates the production of certain M2 markers and some chemokines, thereby facilitating breast cancer cell metastasis (107). CircPVT1 competitively binds to MAVS with RIG-I, thereby inhibiting the aggregation of MAVS and further achieving the negative regulation of type I IFN signaling pathway. Antisense oligonucleotides targeting circPVT1 can resensitize ERα-positive breast cancer cells to endocrine therapy (108). CircPARPBP is markedly elevated in both primary and chemorefractory TNBC specimens and aligns with poor neoadjuvant chemotherapy response. It drives progression and platinum tolerance by recruiting the SNF2-related CBP activator complex to the CCL20 promoter to induce transcription, a feed-forward cascade that entrenches cancer stemness and cements durable therapeutic resistance (109). CircRHBDD1 (4, 5) is markedly enriched in osteotropic breast cancer cells and patient-derived bone metastatic lesions, driving osteoclastogenesis and pre-metastatic niche formation via CCL2. It directly binds the CCL2 transcript—whose levels track with immune infiltration and skeletal spread—and recruits ELAVL1 to stabilize the message and boost translation. Locked nucleic acid (LNA) probes targeting this circRNA-CCL2 interface, termed circRNA–mRNA interaction blockers, potently curb osteoclast activation and bone colonization in preclinical models (110). CircRNAs have been confirmed to regulate TRIM29, which is involved in breast cancer progression (125, 126). TRIM29 has been shown to regulate type I interferon production (127–129), PERK-mediated endoplasmic reticulum stress (130), and inflammasome activation (131). Notably, these three pathways are critically implicated in tumorigenesis, tumor progression, and antitumor immunity (132–134). Circular RNAs have the potential to influence breast cancer tumor immunity and immunotherapeutic outcomes through modulation of TRIM29 expression or activity. This possibility warrants further experimental validation.

Overall, circRNAs serve as modulators within the cytokine-enriched breast cancer immune microenvironment, which reshapes stromal-immune crosstalk, reinforces cancer stemness, and promotes the formation of permissive metastatic niches, ultimately affecting breast cancer progression, metastasis, and therapy resistance. This axis therefore represents a mechanistically defined and actionable target for reversing immune evasion in breast cancer (**Figure 3**).

**Figure 3:**
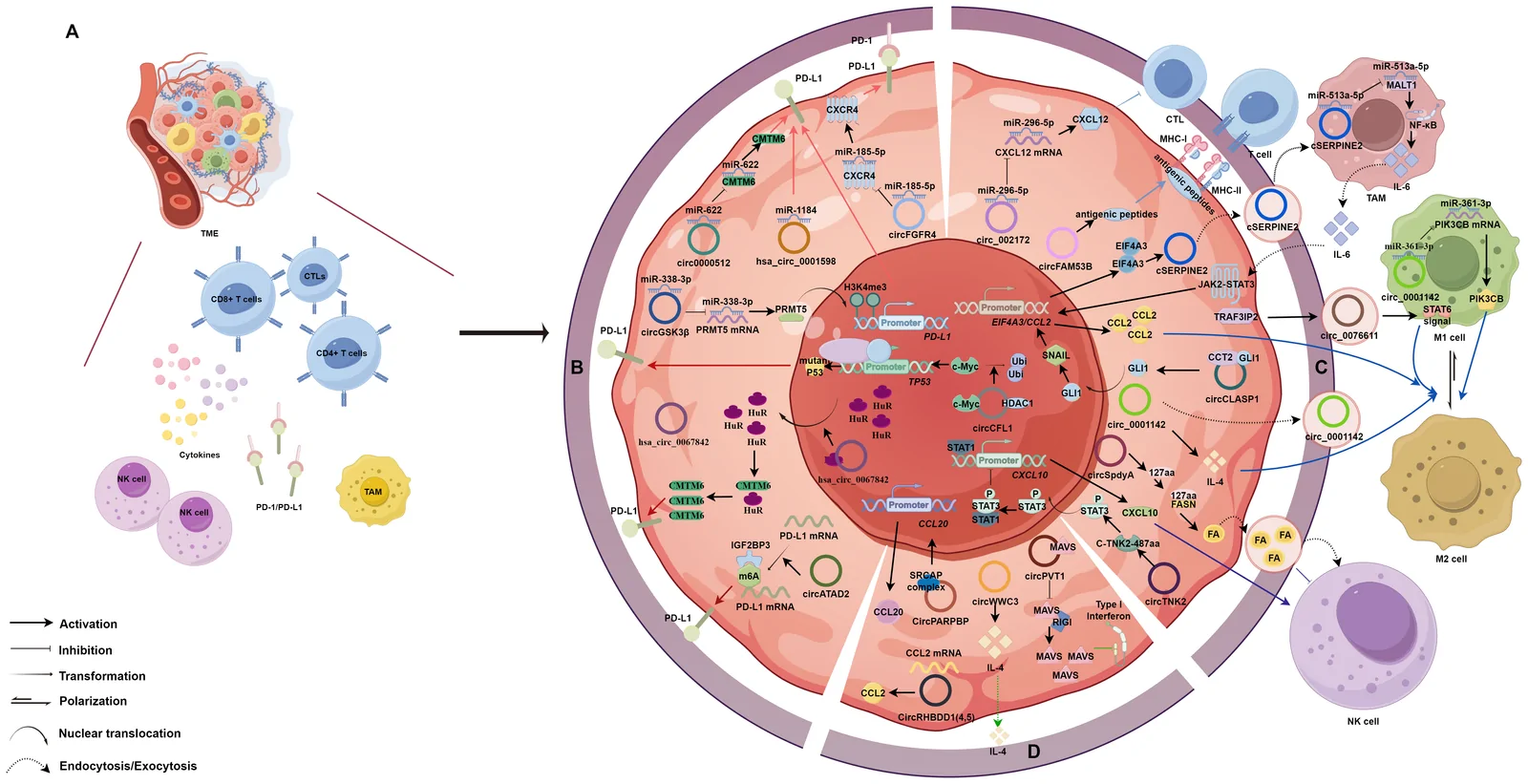
The roles and mechanisms of circular RNAs in breast cancer immunotherapy. **(A)** The Overview of the function of circular RNAs in breast cancer immunotherapy. **(B)** Circular RNAs are involved in the regulation of anti-PD-L1 immunotherapy in breast cancer. **(C)** Circular RNAs affect immune cells in breast cancer. **(D)** Circular RNAs regulate cytokines interacting with immune cells.

## 4 Discussion

### 4.1 Aspects of clinical significance of related circular RNAs

CircRNAs possess high stability due to their unique circular structure, which lays a solid foundation for its application in breast cancer immunotherapy. Research has demonstrated that circRNAs facilitate breast cancer initiation and progression via multiple mechanisms. For example, certain circRNAs function as miRNA sponges, whereas others bind proteins, encode polypeptides, or serve as regulators of gene expression, ultimately influencing key biological processes, including proliferation, apoptosis, and metastasis. Over the past few years, evidence indicates that circRNAs are also key regulatory molecules in the immune system, exerting significant influences on immune regulation, especially playing an important role in modulation of immune checkpoints, immune cells, and immune-related cytokines. Using specific circRNAs as targets for breast cancer immunotherapy has become a powerful means to improve the cure rate and overall survival of breast cancer.

Currently, in preclinical studies, the application of circRNAs in tumor immunotherapy has shown some encouraging results. By constructing circRNA vectors expressing specific immune regulatory factors, immune cells can be effectively activated *in vitro*, enhancing their ability to kill breast cancer cells. Due to their special circular structure, in conjunction with internal ribosome entry sites and open reading frames in some circRNAs, circRNA-based vaccines exhibit superior stability and safety profiles, providing a new strategy for the application of RNA vaccine therapy (135). At the same time, progress has been made in the research into *in vivo* transfer of circRNAs. The application of novel delivery systems such as nano-carriers has significantly improved the enrichment of circRNAs in tumor tissues, providing a guarantee for their role in immunotherapy (136). Designing drugs targeting circRNAs based on small interfering RNAs and antisense oligonucleotides to control circRNA expression levels, thereby shaping the immune system and boosting the effectiveness of breast cancer immunotherapy, has promising prospects. In terms of basic research, future efforts should prioritize further investigation into novel circRNA-mediated immune regulatory mechanisms in breast cancer, identification of additional circRNA targets with therapeutic value, and establishment of a theoretical framework for developing innovative immunotherapeutic approaches. Meanwhile, with the help of gene editing technologies such as the CRISPR-Cas system, circRNAs can be precisely designed and modified to enhance their immune regulatory activity and stability (137). In terms of clinical transformation, optimizing the delivery system of circRNAs is a key link to realize their clinical application. Developing more efficient, safe, and targeted delivery carriers to accurately deliver circRNAs to immune cells can boost therapeutic efficacy and minimize side effects. In addition, combination therapy strategies are also an important development direction in the future (138–140). Many studies have found that some chemotherapy and neoadjuvant chemotherapy drugs can induce immunogenic cell death, thereby recruiting more tumor-infiltrating lymphocytes into the tumor microenvironment, or directly eliminating immunosuppressive cells to remodel the tumor microenvironment (141, 142). Combining circRNA immunotherapy with chemotherapy, neoadjuvant chemotherapy, radiotherapy, and other treatment methods can exert synergistic effects, regulate the tumor microenvironment, and increase the sensitivity of tumors to immunotherapy.

### 4.2 Challenges of circular RNAs in breast cancer immunotherapy

However, circRNA-based approaches in breast cancer immunotherapy still face many challenges. On the one hand, there are methodological shortcomings in the cited experiments, such as excessive reliance on *in vitro* cell experiments and animal models. The functional mechanisms of circRNAs remain incompletely understood, and the specific functions of many circRNAs in the complex immune regulatory network and their upstream and downstream molecular pathways remain to be further explored. On the other hand, how to achieve precise *in vivo* delivery of circRNAs, break through the barriers of the tumor microenvironment, achieve high tumor specificity and promote long-term stable expression, while reducing their potential immunogenicity, is still key issues to be solved. At the same time, due to the existence of immune escape and immunosuppression, the single application of breast cancer immunotherapy is greatly limited, and drug resistance has become a primary barrier hindering the implementation of immunotherapy in breast cancer (143).

Beyond these mechanistic and delivery complexities, the path to clinical translation is further impeded by a constellation of technical and regulatory obstacles that warrant critical evaluation. Firstly, the absence of standardized, high-throughput detection protocols for circRNAs—particularly for low-abundance isoforms in biofluids—hinders accurate biomarker validation and pharmacokinetic profiling. Current methodologies, ranging from RNA sequencing to quantitative polymerase chain reaction, lack uniformity in normalization and quantification, complicating cross-study comparisons and clinical diagnostics.

Secondly, while circRNAs possess inherent stability, their pharmacological behavior introduces unique pharmacokinetic profiling and safety concerns. The potential for off-target effects, arising from the promiscuous binding of circRNAs to unintended RNAs or protein targets, remains inadequately characterized. Furthermore, the long-term persistence of circRNAs, while advantageous for sustained expression, raises questions regarding dose-limiting toxicity and the potential for insertional mutagenesis, especially when employing viral vectors for delivery. The immunogenicity profile of circRNAs themselves is also double-edged; although generally low, certain circRNAs can trigger innate immune sensors (e.g., PKR, RIG-I), necessitating rigorous immunotoxicity screening (144, 145).

Thirdly, the profound tumor heterogeneity characteristic of breast cancer poses a significant barrier to the efficacy of circRNA therapeutics. A circRNA target identified in one molecular subtype may be irrelevant or even deleterious in another, demanding a precision medicine approach that is currently lacking. This heterogeneity also underlies the rapid emergence of therapeutic resistance, as clonal evolution can select for cells that bypass the targeted circRNA pathway (146).

Finally, the regulatory landscape for circRNA-based therapeutics remains nascent and fragmented. Unlike small molecules or monoclonal antibodies, circRNAs represent a novel class of biologics without established regulatory pathways or standardized guidelines for synthesis, purification, and quality control. The paucity of large-scale, well-designed clinical trials—especially those combining circRNA modulators with existing immune checkpoint inhibitors—leaves a critical void in our understanding of their true clinical efficacy and safety profile in breast cancer patients. Addressing these multifaceted challenges through collaborative efforts in assay development, delivery system optimization, and regulatory science will be paramount to bridging the gap between promising preclinical findings and tangible clinical benefits.

## 5 Conclusion

CircRNAs affect breast cancer immunotherapy by engaging in the modulation of immune checkpoints including PD-1/PD-L1 and shaping the activity of immune cells and cytokines within the tumor microenvironment, which deserves further research in the future. The inherent structural stability, tissue specificity, and diverse regulatory capabilities of circRNAs confer them with non-negligible translational potential in the field of breast cancer immunotherapy. In the future, circRNA-targeting therapeutic strategies require further research for advancement from preclinical studies to clinical applications. Leveraging technologies such as high-throughput sequencing, multi-omics, RNA modification techniques, and precision delivery, it is expected that off-target effects will be reduced, biosafety will be improved, and delivery efficiency will be enhanced. Ultimately, circRNAs hold promise for opening up novel strategies and avenues for precision immunotherapy in breast cancer.
